# The effect of environmental sustainability orientations and entrepreneurial orientations on the performance of greenhouses

**DOI:** 10.1038/s41598-024-52062-y

**Published:** 2024-01-24

**Authors:** Ahmad Yaghoubi Farani, Saeid Karimi, Mina Sajedi, Pouria Ataei

**Affiliations:** 1 Department of Agricultural Extension and Education, Bu-Ali Sina University, Hamadan, Iran; 2 Department of Agricultural Extension and Education, Bu-Ali Sina University, Hamadan, Iran; 3 Tarbiat Modarres University, Tehran, Iran

**Keywords:** Sustainability, Environmental impact

## Abstract

This research aimed to explore the effect of environmental sustainability and entrepreneurial orientations on the performance of greenhouses in Iran. It is a sort of descriptive-correlational research conducted by the survey methodology. The data collection instrument was a questionnaire whose validity was checked by a panel of entrepreneurship and environment experts, and its reliability was estimated by conducting a pilot study and calculating Cronbach’s alpha (α = 0.88–0.91). The statistical population was composed of all greenhouse units in Mahallat County in Markazi province, Iran (N = 405). The sample size was determined to be 197 greenhouses by Krejcie and Morgan’s table. The independent variables included environmental sustainability orientations (including the dimensions of environmental knowledge and awareness, practices, and commitment) and entrepreneurial orientation of greenhouse managers (including the dimensions of innovation, proactiveness, and risk-taking), and the dependent variable was the performance of greenhouses (including the dimensions of increasing customers, increasing sales, and increasing profitability). The results of structural equation modeling showed that the dimensions of environmental sustainability orientations and entrepreneurial orientations of the greenhouse managers were decisive factors in the performance dimensions of the greenhouses. Furthermore, the entrepreneurial orientations had a positive and significant effect on the environmental sustainability orientations.

## Introduction

Sustainable entrepreneurship is an important issue that has long been a key pillar of business development approaches, especially in the agricultural sector. In other words, sustainable entrepreneurship is one of the likely ways for the management of environmental degradation and one of the main dimensions of the agricultural sector, so sustainable agriculture can be developed by considering the potential of sustainability and creating a positive attitude toward the use of sustainable entrepreneurship^1,2^. Although there are various perspectives on sustainable entrepreneurship, recent research on this issue has attempted to integrate environmental and social aspects^3,4^ and views sustainable entrepreneurship as a sort of entrepreneurship that includes the social, economic, and environmental concerns of the stakeholders^5,6^. Accordingly, the innovative behavior of businesses that regard environmental or social issues as a major competitive advantage has been revealed^7^. Johnson and Hörisch^8^ conceive this type of entrepreneurship as a process to achieve sustainable development by discovering, evaluating, and utilizing opportunities and creating value that brings about economic prosperity, social solidarity, and environmental conservation.

According to George and Marino^9^ ustainable entrepreneurial orientation is a dynamic, adaptive, and innovative skill with an external orientation and implies a general strategic orientation at the business level^10^ and shows a business’s readiness to adopt preventive, creative, and risk-taking processes, functions, and behaviors^11,12^ in order to achieve sustainable development^1^. On the other hand, due to the environmental issues and concerns in the context of businesses, sustainable entrepreneurial orientation has also been considered from the perspective of environmental sustainability because it is conceptually perceived as a commercial attitude showing a business philosophy for doing environmentally sustainable trade. In this concept, a business develops its environmental sustainability orientation and trade goals by incorporating environmental concerns into its culture, decision-making, strategy, and trade operations and interacting with stakeholders to minimize the adverse environmental impacts of its business^13,14^.

It is vital to consider environmental issues in the context of agricultural businesses, especially greenhouses because the escalation of greenhouse production in recent years has needed the application of modern technologies that require the use of fertilizers and pesticides whose persistent use over years would have destructive effects on the natural resources and environment. In this regard, over 25,000 greenhouses have been constructed in Iran to achieve fundamental developments in the agricultural sector. Greenhouse cultivation has extensively been expanded for off-season production and optimal use of water and soil resources, especially the use of small land parcels and facilities in rural areas and suburbs of overcrowded areas that lack adequate land and water. In the meantime, excessive use of chemical inputs is quite prevailing in greenhouse cultivation, casting doubts on the sustainability of these units and challenging the accomplishment of sustainable agriculture^15^.

So, given the multifaced role of greenhouses in agriculture and the environment, accomplishing sustainability in all its dimensions seems to be an undeniable fact. In addition, adopting environmental sustainability and entrepreneurial orientations by greenhouse managers can be an effective factor in improving their performance in addition to influencing environmental conditions positively, as research like Roxas et al.^16^ has shown that environmental sustainability orientation provides a mechanism for businesses to exploit their entrepreneurial orientation for performance improvement. Accordingly, this research aimed to explore the effect of environmental sustainability orientation and entrepreneurial orientation on the performance of greenhouses in Iran. The research highlights the role of environmental sustainability orientation and entrepreneurial orientations in enhancing greenhouse performance and discusses how greenhouses can be environmentally more sustainable. We can ratiocinate that entrepreneurial orientation forms part of the greenhouses’ strategic and dynamic assets that can nurture and sustain their strategic orientations toward environmental sustainability. Also, the financial benefits of adopting environmentally sustainable business practices by greenhouses in Iran remain debatable although environmental sustainability orientation itself can be a dynamic asset that can potentially endow a greenhouse with sustainable competitive advantage. Furthermore, the results of this study add to the scant literature on the conduct and performance of greenhouse in the face of increasing pressure to become environmentally sustainable. Thus, greater insights into how entrepreneurial orientation can lead more greenhouses to become more environmentally sustainable should be valuable for managers of greenhouses and policy makers and should inform the design of public policy in developing countries.

### Entrepreneurial orientation and environmental sustainability orientation in businesses

Entrepreneurship is a challenging field of research that has drawn the attention of many researchers, and its common theories are based on the discovery and exploitation of economic opportunities through entrepreneurial orientation^17^. However, most research in businesses that have focused on entrepreneurial orientation has tried to answer the question as to how entrepreneurship can be applied^18^. However, entrepreneurial orientation can be considered a management attitude toward strategic decision processes that provides businesses with a basis for entrepreneurial decisions and measures^19^ and is theoretically useful for enterprises^18^.

The concept of entrepreneurial orientation encompasses the processes at the level of businesses, methods, and decision-making style^16^ as well as the strategic orientation of an entrepreneurship-oriented business^20^. Entrepreneurial orientation is a multifaceted construct that includes innovation, proactiveness, and risk-taking^21^. Innovation represents the intention of a business for testing, supporting new ideas, and avoiding fixed procedures^22^. A high rate of technological innovations and/or product market can be used by a business to pursue new opportunities^23^. Proactiveness implies the intention to predict future needs and make changes in the operational environment to pioneer new methods and techniques^24^, thereby gaining a competitive advantage against competitors^23^. Risk-taking is accompanied by the intention to assign more resources to projects that may have high failure costs^16^.

On the other hand, poor environmental standards in many developing countries, which are mostly attributed to their undeveloped institutional environments, are a source of environmental concerns in many countries^25^. Consequently, businesses pay more attention to environmental sustainability because environmental sustainability orientation as an integrated component of businesses’ strategies is regarded as an outcome of strong entrepreneurial orientations of business owners and managers^26^. In other words, orientation towards environmental sustainability is a strategic orientation that points to an organization’s position and extensive arrangements for pursuing a path that shapes the organization’s continuous model as a result of interaction with its trade environments^16^.

Entrepreneurial orientations of businesses refer to their strategic position for integrating environmental considerations with other business strategies. Indeed, it shows the intentional strategies of a business for changing its organizational system, structural processes, and activities to alleviate the negative effects of the business on the environment^16^. Conceptually, it is rooted in common concepts that enterprises need to integrate environmental concerns with their culture, decisions, strategies, and commercial operations and their interactions with different stakeholders^27^. These orientations are like a strategic structure at the business level that covers extensive perceptions of organizational awareness, participation, and commitment to environmental sustainability-related issues, activities, and programs^28,29^.

### Effect of entrepreneurial orientation and environmental sustainability orientation on business performance

Based on the Natural Resource-Based View (NRBV), in the highly uncertain environment, firms strive to seize business opportunities in order to establish sustainable competitive advantages and enhance performance^30,31^. Hart^30^ suggests that firms can develop their strategic capabilities to address problems of environmental pollution, product stewardship and sustainable development, while at the same time gaining competitive advantage and increasing economic performance^16,32^. However, some scholars suggest that companies may incur higher operating expenses by implementing environmentally sustainable practices in their operations, which can negatively impact their financial performance^16^. The integration of eco-friendly business practices can lead to increased costs due to the need for new technologies, equipment, and processes. These expenses can have a direct impact on the bottom line, potentially reducing profits^16^. However, this study was focused on two strategic capabilities namely entrepreneurial orientation and environmental sustainability orientation. According to Roxas et al.^16^, these two capabilities can result in a sustainable competitive advantage as they are strategic resources that are valuable, rare, inimitable and non-substitutable, which can set firms apart in terms of competition^32^.

Regarding the effect of the personal dimensions of entrepreneurial orientation, including innovation, proactiveness, and risk-taking, previous research has shown that each dimension can influence performance positively^16,23^. Innovative companies can exhibit extraordinary economic performance by building and introducing new products and technologies and can even be considered economic growth engines^33^. Proactive companies can control the market by controlling the distribution channels and having their brand names recognized^20^. Research shows that while tested and real strategies may create above-average performance, risky strategies may bring more profit in the long run through the success of some projects even though some projects may fail^34^. Entrepreneurial orientation increases organizational activity and tendency toward risks and innovation^35,36^. Consequently, entrepreneurial orientation may be regarded as a prerequisite for innovative performance^37^. The innovative performance of a business includes product and process innovation, which are close and interrelated consequences^38^ and constitute a very sophisticated process that encompasses all performances of the business^39,40^.

Various studies have pointed to and confirmed the effect of entrepreneurial orientations on the performance of different businesses^41–43^. For example, Forcadell and Úbeda^44^ established that entrepreneurial orientations improve business performance indirectly and nonlinearly. Alam et al.^45^ concluded that entrepreneurial orientations promote business performance. They state that entrepreneurs cannot ignore this element in their activities, especially in business viability.

Entrepreneurial orientation can be a dynamic asset, specifically in the field of environmental sustainability, and can potentially create sustainable advantages for businesses^46^. Although it may impose heavy costs for businesses in its early steps, its long-term benefits will obviously outweigh the costs of the strategic position^47^. Aftab et al.^48^ report that entrepreneurial orientation can reinforce business performance and sustainable development methods. Frare and Beuren^49^ state that green innovation plays a key role in the performance and promotes full mediation between green entrepreneurial orientation and active sustainable strategies with environmental performance. They enumerate two ways to achieve high performance. Innovation is a core requirement for both ways, but entrepreneurial orientation is a complementary requirement. Dias et al.^50^ argue that entrepreneurial and environmental orientations are the link in the relationship between natural resources and performance, showing strong internal responses to agricultural occupations with natural environmental constraints.

Continuous approval and integration of environmental concerns with main business activities will lead to finding creative ways to deal with the negative impacts of the business on the natural environment^51^. Indeed, although the adoption of environmental sustainability orientations and the design and fulfillment of environmentally compatible methods can have negative effects at the low level by significantly increasing the operational costs of the occupations, these strategic capabilities can, in contrast, be a potential source of competitive advantage because they are strategic resources that are valuable, rare, unique, and non-exchangeable and distinguish a business from its competitors^52^. In this regard, Hart and Dowell^53^ propose that environmental sustainability orientation can provide a company with competitive advantages and the company can use cost reduction related to the reduction of wastes and pollutant emissions, as well as the increased legitimacy of its stakeholders at the local and international levels because the environmental aspect typically tries to reduce the use of valuable environmental resources, reduce the generation and use of harmful material, and prevent environmental pollution and waste production^54,55^.

In a study on the effect of entrepreneurial and environmental sustainability orientations, Roxas et al.^16^ emphasized that both orientations improve business performance. On the other hand, environmental sustainability orientations are influenced by entrepreneurial orientations. They also reported that environmental sustainability orientations mediate the effects of entrepreneurial orientations on the performance of small businesses. Aragón-Correa et al.^56^ who studied the relationship between environmental strategy and business performance found a positive and significant relationship between preventive and environmentally-friendly methods and the performance of small and medium-sized enterprises. Other researchers have also supported the significance of environmental sustainability orientations and their effect on business performance^33,57–63^ and the effect of entrepreneurial orientations on environmental sustainability orientations^1,49,50,64,65^.

**Figure 1:**
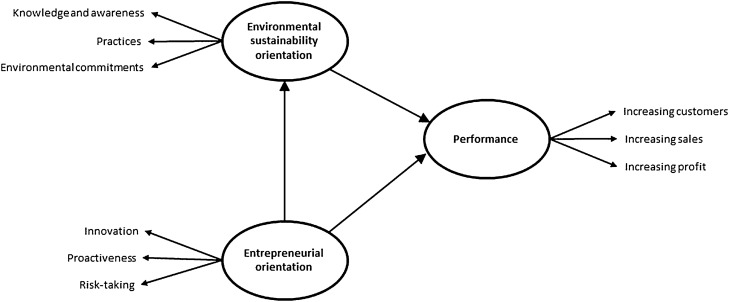
The conceptual framework of the study.

The literature review shows that there has been a logical relationship between entrepreneurial orientations, environmental sustainability orientations, and business performance. According, the conceptual framework of the research was developed as depicted in Fig. 1 and the following hypotheses were considered to achieve the research goals.

- Hypothesis 1: Environmental sustainability orientation influences the performance of greenhouses positively and significantly.
- Hypothesis 2: Entrepreneurial orientation influences the performance of greenhouses positively and significantly.
- Hypothesis 3: Entrepreneurial orientation influences the environmental sustainability orientation of greenhouses positively and significantly.
- Hypothesis 4: Environmental sustainability orientation mediates the relationship between entrepreneurial orientation and the performance of greenhouses.

## Methodology

The research is an applied study in goal and a descriptive study in data analysis method. Data were collected by the survey method. The research domain included all active greenhouses in Mahallat County in Markazi province, Iran. The research population was composed of these greenhouses estimated to be 405 units based on the statistics provided by the Agriculture Organization of this province (70 units for summer crops and 335 units for floriculture). The sample was taken by stratified random sampling with proportional allocation. According to Krejcie and Morgan’s^66^ table, the sample size was estimated at 197 units (30 summer crop greenhouses and 167 floriculture greenhouses). Data were collected using standard questionnaires. The dependent variable was the performance of the greenhouse businesses, which was evaluated in three dimensions of increasing customers, increasing sales, and increasing profit^16^. The independent variables included environmental sustainability orientation (knowledge and awareness, practices, and environmental commitment)^16^ and entrepreneurial orientation (innovation, proactiveness, and risk-taking)^67^.

The face and content validity of the research instrument was confirmed by a panel of entrepreneurship and agriculture experts, and its construct validity was found to be at an optimal level. The reliability of the questionnaire was confirmed by calculating Cronbach’s alpha and composite reliability (CR) in a pilot study. Cronbach’s alpha and CR for all constructs confirmed their compatibility. The variables were measured on a five-point scale (from 1 = very low to 5 = very high). Data were analyzed by SPSSwin26 and Smart PLS software packages. Also, the data were analyzed by calculating means, coefficients of variations, and structural equation modeling.

### Ethics approval and consent to participate

Because of the retrospective, cross-sectional and anonymous character of this study, the need for ethics approval and informed consent was waived by the Research and Ethics Committee of the Department of Agricultural Extension and Education, Faculty of Agriculture, Bu-Ali Sina University, Hamedan, Iran.

We confirm that all methods were carried out in accordance with relevant guidelines and regulations. Written informed consent was obtained from all individual participants included in the study.

## Results

### Demographic characteristics of greenhouse managers

According to the results of farmers’ demographic characteristics of greenhouse managers, 93.9 percent were male and 6.1 percent were female. The lowest age was 25 years and the highest was 51 years with a mean age of 36.45 years. In terms of the educational level, 67 greenhouse managers, which was the highest frequency (34%), had bachelor’s degrees and 27 managers (13.7%) had under-diploma education. Also, 17.8 percent had diplomas, 19.3 percent had associate’s degrees, and 15.2 percent had master’s degrees or higher. Regarding the frequency of attending entrepreneurial and marketing educational courses, 60 participants had attended one course, 99 participants had attended two courses, and 18 participants had attended three courses or more. However, 20 managers had attended no educational courses. Furthermore, the participants had been working in the greenhouse for, on average, 12.33 years. Also, 115 participants (58.4%) had used bank credits while 82 participants (42.6%) hadn’t.

### Evaluation of the measurement model

The reliability of the questionnaire was evaluated by calculating Cronbach’s alpha and CR. The results indicated that value of CR and Cronbach’s alpha are more than 0.5 and 0.7 for all variables, respectively (Table 1). Furthermore, the construct validity was assessed using the average variance extracted (AVE) which is proper (values were higher than 0.6 for each variables). As shown in Table 1, Heterotrait-monotrait ratio (HTMT) and Fornell-larcker (FL) criteria also were estimated to investigate discriminant validity. The results of discriminant validity illustrated that the items selected to measure the variables of conceptual framework had the proper discriminant validity.

**Table 1:** Reliability and validity of the research variables.

Construct	1	2	3	Α	CR	AVE
1. Environmental orientation	1			0.90	0.90	0.75
2. Entrepreneurial orientation	0.686	1		0.92	0.92	0.80
3. Performance of greenhouses	0.662	0.760	1	0.90	0.90	0.75

The face and content validity of the research instrument was confirmed by a panel of entrepreneurship and agriculture experts, and its construct validity was found to be at an optimal level. The reliability of the questionnaire was confirmed by calculating Cronbach’s alpha and composite reliability (CR) in a pilot study. Cronbach’s alpha and CR for all constructs confirmed their compatibility (Table 1). The convergent validity of the constructs was investigated by average variance extracted (AVE), which was found to be in the range of 0.75–0.80, showing that all values were close to or higher than the acceptable level of 0.5. The HTMT approach was employed to assess the divergent validity^68^. Based on the results (Table 1), all HTMT values were lower than the acceptable level of 0.85, reflecting the divergent validity of the research scales.

### Evaluation of the structural model

**Figure 2:**
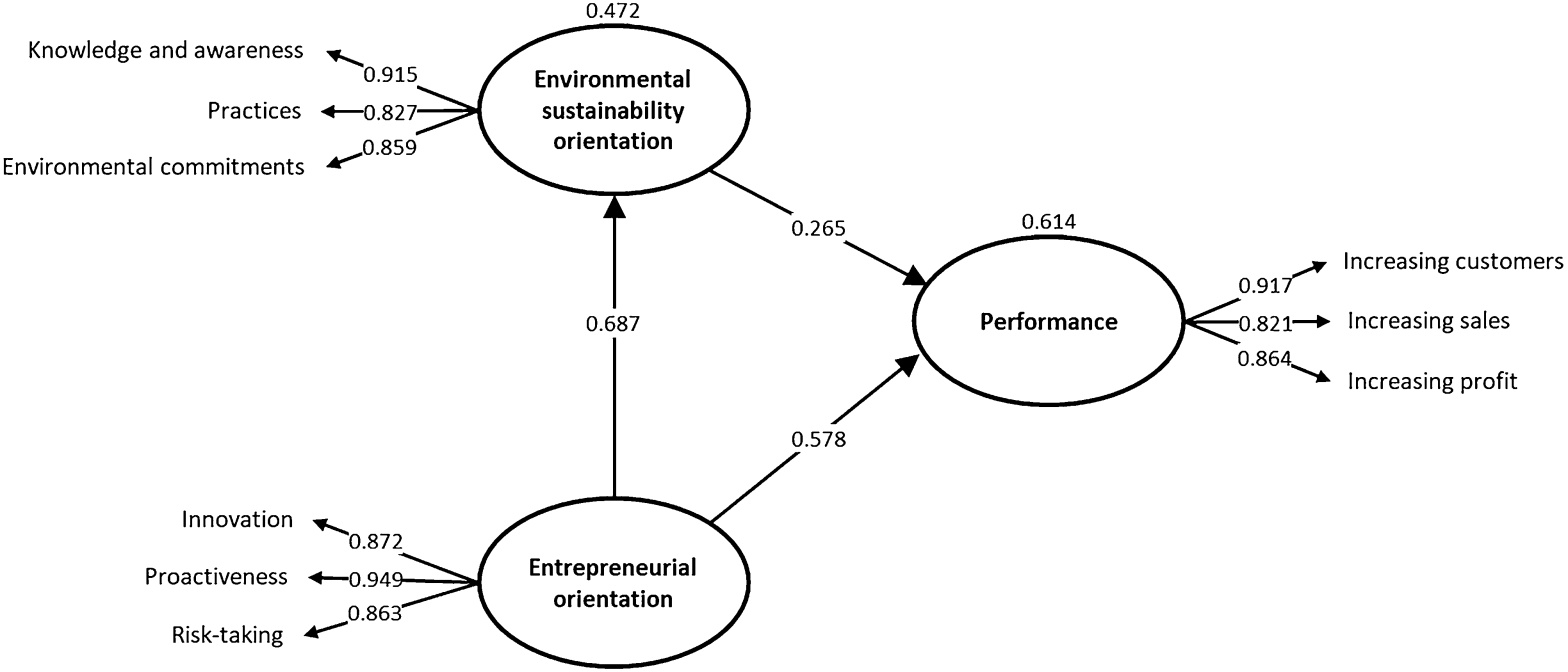
The results of causal relationships between the research variables.

The results showed that the environmental sustainability orientation of the greenhouses influenced their performance positively and significantly (β = 0.26, *P* < 0.05). This supports hypothesis 1. It can be argued that if knowledge and awareness of environmental issues, environmental practices, and commitment to environmental issues are promoted, greenhouse performance will be increased in customer, sales, and profitability aspects. The results also revealed the positive and significant effect of the entrepreneurial orientation of the greenhouse managers on greenhouse performance (β = 0.57, *P* < 0.05), supporting hypothesis 2. In this regard, it can be claimed that as the tendency of greenhouses toward innovation, proactiveness in entrepreneurial orientation, and risk-taking increases, they exhibit better performance. Finally, it was found that the entrepreneurial orientation of the greenhouse managers had a positive and significant effect on the environmental orientation of the greenhouses (β = 0.68, *P* < 0.05). This confirms hypothesis 3 (Table 2, Fig. 2). It can be asserted that greenhouse managers’ entrepreneurial orientation improves knowledge and awareness of environmental issues, increases environmental practices, and enhances commitment to environmental issues in greenhouses.

**Table 2:** Hypothesis testing direct and indirect effects.

Hypothesis	Relationship	*β*	t-values	*p*-values	BCI LL	BCI UL	Decision
Direct effects							
H1	Environmental sustainability orientation → Performance	0.265	3.883	0.000	0.126	0.392	Supported
H2	Entrepreneurial orientation → Performance	0.578	8.750	0.000	0.407	0.633	Supported
H3	Entrepreneurial orientation → Environmental sustainability orientation	0.687	17.744	0.000	0.559	0.693	Supported
Indirect effects							
H4	Entrepreneurial orientation → Environmental sustainability orientation→ Performance	0.167	3.926	0.000	0.08	0.251	Supported
Total effects							
	Entrepreneurial orientation → Performance	0.745	18.845	0.000	0.615	0.766	

*β:* standardized path coefficient; *CI* confidence interval; *f*^*2*^ effect size, *BCI LL* Bootstrapped Confidence Interval Lower level; *BCI UL* Bootstrapped Confidence Interval Upper level.

The coefficient of determination (R^2^) was estimated at 0.614 for the performance of the greenhouses. It implies that 61.4% of the variance in their performance is related to the entrepreneurial orientation of the greenhouse managers and the environmental orientation of the greenhouses. As well, R^2^ was estimated at 0.472 for the environmental orientation of the greenhouses, meaning that 47.2 percent of its variance is predicted by the entrepreneurial orientation of their managers.

To test the mediation hypothesis, the procedure of mediation analysis was conducted in PLS using Nitzl et al.’s^69^ guideline. According to Nitzl et al.^69^ “testing the indirect effect a × b provides researchers with all information for testing mediation”; here “a” refers to the path between the independent variable and the mediator variable, while “b” represents the path between the mediator variable and the dependent variable. The significance test of the indirect effect showed that the indirect relationship between entrepreneurial orientation and performance via environmental sustainability orientation (*β* = 0.167*, CI* = *[0.08, 0.251]*) was significant because zero was not included in the 95% confidence intervals. Therefore, environmental sustainability orientation partially mediated the relationship between entrepreneurial orientation and performance, supporting H4.

## Discussion

This research mainly aimed to evaluate the effects of the entrepreneurial orientation of greenhouse managers on the environmental sustainability orientation of the greenhouses and its implications for their performance in Iran. The research provides strong evidence for the positive effect of the greenhouse managers’ entrepreneurial orientation on the development of environmental sustainability orientation in greenhouses. It was also revealed that these two strategic orientations contributed to the performance of the greenhouses positively. These findings cast doubts on common sense that agricultural activities (especially greenhouses) in developing countries lack the financial and managerial resources required for making them environmentally sustainable with no adverse impact on their performance. In other words, the research provides empirical evidence that despite resource limitations, greenhouses can participate in environmentally sustainable commercial methods if their managers have entrepreneurial orientations. This is contrary to the view that small businesses are generally unable to pursue environmentally sustainable methods as compared to larger businesses^16,70^. The adoption of environmentally sustainable methods by small businesses to a lesser extent than larger businesses may be related to Varquez the limitations of resources and their less systematic structure^71,72^. Our results reveal that the entrepreneurial orientation of greenhouses contributes to tackling their inherent limitations in developing environmental sustainability orientation. This finding agrees with the results reported by some researchers^1,16,33,45,48^. They have also concluded that the entrepreneurial orientation of businesses promotes their performance.

A more active, innovative, and risk-taking attitude is required for the development of environmental sustainability orientation in greenhouses. This specific finding can provide a more precise understanding of how strategic factors like risk-taking, proactiveness, and innovation may better describe why some agricultural businesses have stronger intentions toward integrating environmental sustainability with their decision-making and commercial activities. Previous studies have revealed that a business should first look at what it itself does before identifying and implementing sustainability practices such as reducing the use of chemical pesticides and controlling pollution^16,49,61,63,73,74^. Although the environmental sustainability orientation of greenhouses may at first be guided by conformity with national regulations^25,75^, the greenhouses that have successfully developed a strong strategic orientation toward sustainability are more capable of continuous learning and innovation.

The results provide empirical evidence for the fundamental principle of the natural resource-based view (NRBV)^30^ that a business can achieve environmental sustainability alongside profitability. Based on the NRBV, this study has indicated small businesses can improve their performance by embracing sustainability practices through leveraging their entrepreneurial orientation. Therefore, the study has made a substantial contribution to the theory and expanded the literature from the perspective of developing economies (“Supplementary Information”).

## Conclusions

In general, this research revealed the important role of the greenhouse managers’ entrepreneurial orientation in shaping greenhouses’ environmental sustainability orientation in Iran and the effect of these two variables on the performance of the greenhouses. The results highlight the crucial fact that despite the inherent limitations of resources and other structural limitations of greenhouses in Iran, they can foster their strategic orientation to integrate environmental sustainability in their businesses, thereby responding to this new challenge of greenhouse development. The results show that an entrepreneurial position based on proactiveness, risk-taking, and innovation plays a key role in coping with these limitations in developing a strategic orientation toward environmental sustainability in greenhouses. Entrepreneurial attitude can empower greenhouse development in Iran to tackle their limited resources by innovation and discover new ways for doing their agricultural business more environmentally friendlier despite ambiguities in building greenhouses, which is replete with institutional gaps as is the characteristic of emerging or developing economies. Finally, a strategic orientation that is focused on environmental sustainability and is consistent with the distinctive configuration of greenhouses may be helpful for their performance. The results support the argument that greenhouses can be environmentally sustainable in spite of their limited size and resources. Consequently, this research highlights the effect of the entrepreneurial orientation of greenhouse managers and their environmental sustainability orientation on the improvement of their performance and contributes to the current discussion on how governments and agriculture organizations in developing countries can motivate greenhouses to be environmentally more sustainable.

According to the results, it is recommended to financially and scientifically support producers that have a stronger tendency toward innovation, proactiveness, and risk-taking in greenhouses. Furthermore, the procedure of issuing licenses required for greenhouse construction should be facilitated for people who are committed to observing environmental sustainability requirements in their businesses. Also, the identification of weaknesses and strengths of plans and efforts to increase environmental awareness, practices, and commitment can be a proper approach.

There may be some limitations to any study. This study also faced limitations like other researches. One of the limitations was measuring the performance of greenhouses based on the Likert scale. If the performance of greenhouses is measured based on economic or quantitative indicators, a proper understanding of this variable can be found. In future studies, different indicators can be used to measure the performance of greenhouse. Furthermore, the greenhouses in this study were selected from a single county. Different county or province may have differences in environmental orientation, entrepreneurial orientation, and performance. Future research could obtain greenhouses from multiple counties or provinces to identify and compare these differences.

### Supplementary Information

Supplementary Information.

## Data Availability

The data that support the findings of this study are available from Bu-Ali Sina University but restrictions apply to the availability of these data, which were used under license for the current study, and so are not publicly available. Data are however available from the authors upon reasonable request and with permission of the corresponding author.

## References

[1] Andersén J (2022). An attention-based view on environmental management: The influence of entrepreneurial orientation, environmental sustainability orientation, and competitive intensity on green product innovation in Swedish small manufacturing firms. Organ. Environ..

[2] Mamani WC, Manrique GML, Madrid SDPC, Herrera EE, Acosta DB, Rivas-Diaz RR, Ramos FSS (2022). The role of entrepreneurship and green innovation intention on sustainable development: Moderating impact of inclusive leadership. AgBioForum.

[3] Kyrö P (2001). To grow or not to grow? Entrepreneurship and sustainable development. Int. J. Sustain. Dev. World Ecol..

[4] Panyasing S, Yongvanit S, Purnomo EP, Tham I, Aim S (2022). The government policy on the organic rice farming groups embracing sustainable agricultural production: Evidence in Thailand. AgBioforum.

[5] De Palma R, Dobes V (2010). An integrated approach towards sustainable entrepreneurship—Experience from the TEST project in transitional economies. J. Clean. Prod..

[6] Dinh HP, Vo PH, Pham DN, Ngo TQ (2022). Factors affecting farmers' decisions to participate in agricultural tourism activities: A case study in the Mekong delta, Vietnam. AgBioForum.

[7] Al-Qahtani M, Zguir MF, Al-Fagih L, Koç M (2022). Women entrepreneurship for sustainability: investigations on status, challenges, drivers, and potentials in Qatar. Sustainability (Switzerland).

[8] Johnson MP, Hörisch J (2022). Reinforcing or counterproductive behaviors for sustainable entrepreneurship? The influence of causation and effectuation on sustainability orientation. Bus. Strateg. Environ..

[9] George BA, Marino L (2011). The epistemology of entrepreneurial orientation: Conceptual formation, modeling, and operationalization. Entrep. Theory Pract..

[10] Engelen A, Kube H, Schmidt S, Flatten TC (2014). Entrepreneurial orientation in turbulent environments: The moderating role of absorptive capacity. Res. Policy.

[11] Matsuno K, Mentzer JT, Özsomer A (2002). The effects of entrepreneurial proclivity and market orientation on business performance. J. Mark..

[12] Yaghoubi Farani A, Karimi S, Izadi N, Ataei P (2019). Effect of virtual social networks on entrepreneurial behavior of agricultural students. Appl. Econ..

[13] Zwetsloot GIJM, van Marrewijk MNA (2004). From quality to sustainability. J. Bus. Ethics.

[14] Walter, S., Boden, B., Günter, K., Paul, B., Lukas, F. & Lea, H. Analyze the relationship among information technology, precision agriculture, and sustainability. *J. Commer. Biotechnol.* **27**(3) (2022).

[15] Van Thanh N, Yapwattanaphun C (2015). Banana farmers’ adoption of sustainable agriculture practices in the Vietnam uplands: The case of Quang Tri Province. Agric. Agric. Sci. Procedia.

[16] Roxas B, Ashill N, Chadee D (2017). Effects of entrepreneurial and environmental sustainability orientations on firm performance: A study of small businesses in the Philippines. J. Small Bus. Manag..

[17] Runyan R, Droge C, Swinney J (2008). Entrepreneurial orientation versus small business orientation: What are their relationships to firm performance?. J. Small Bus. Manag..

[18] Duane Ireland R, Webb JW (2007). A cross-disciplinary exploration of entrepreneurship research. J. Manag..

[19] Richard OC, Barnett T, Dwyer S, Chadwick K (2004). Cultural diversity in management, firm performance, and the moderating role of entrepreneurial orientation dimensions. Acad. Manag. J..

[20] Wiklund J, Shepherd D (2003). Knowledge-based resources, entrepreneurial orientation, and the performance of small and medium-sized businesses. Strateg. Manag. J..

[21] Wiklund J (1999). The sustainability of the entrepreneurial orientation-performance relationship. Entrep. Theory Pract..

[22] Lumpkin GT, Dess GG (1996). Clarifying the entrepreneurial orientation construct and linking it to performance. Acad. Manag. Rev..

[23] Wiklund J, Shepherd D (2005). Entrepreneurial orientation and small business performance: A configurational approach. J. Bus. Ventur..

[24] Lee C, Lee K, Pennings JM (2001). Internal capabilities, external networks, and performance: A study on technology-based ventures. Strateg. Manag. J..

[25] Dangelico R, Pujari D (2010). Mainstreaming green product innovation: Why and how companies integrate environmental sustainability. J. Bus. Ethics.

[26] Spence M, Gherib JBB, Biwolé VO (2011). Sustainable entrepreneurship: Is entrepreneurial will enough? A north–south comparison. J. Bus. Ethics.

[27] Linnenluecke MK, Griffiths A (2010). Corporate sustainability and organizational culture. J. World Bus..

[28] Nawi NC, Mamun AA, Daud RRR, Nasir NAM (2020). Strategic orientations and absorptive capacity on economic and environmental sustainability: A study among the batik small and medium enterprises in Malaysia. Sustainability (Switzerland).

[29] Wang CH (2020). An environmental perspective extends market orientation: Green innovation sustainability. Bus. Strategy Environ..

[30] Hart SL (1995). A natural-resource-based view of the firm. Acad. Manag. Rev..

[31] Rehman SU, Kraus S, Shah SA, Khanin D, Mahto RV (2021). Analyzing the relationship between green innovation and environmental performance in large manufacturing firms. Technol. Forecast. Soc. Change.

[32] Porter ME (1985). Technology and competitive advantage. J. Bus. Strategy.

[33] Adomako S, Amankwah-Amoah J, Danso A, Konadu R, Owusu-Agyei S (2019). Environmental sustainability orientation and performance of family and nonfamily firms. Bus. Strategy Environ..

[34] McGrath RG (2001). Exploratory learning, innovative capacity, and managerial oversight. Acad. Manag. J..

[35] Altantsetseg P, Dadvari A, Munkhdelger T, Lkhagvasuren GO, Moslehpour M (2020). Sustainable development of entrepreneurial orientation through social drivers. Sustainability (Switzerland).

[36] Munawar MM, Hurriyati R, Disman D, Gaffar V (2023). Improving business performance through entrepreneurial orientation, product innovation, and co-creation value. Int. J. Innov. Res. Sci. Stud..

[37] Baker WE, Sinkula JM (2009). The complementary effects of market orientation and entrepreneurial orientation on profitability in small businesses. J. Small Bus. Manag..

[38] Butkouskaya V, Llonch-Andreu J, Alarcón-del-Amo MDC (2020). Entrepreneurial orientation (EO), integrated marketing communications (IMC), and performance in small and medium-sized enterprises (SMEs): Gender gap and inter-country context. Sustainability (Switzerland).

[39] Alegre J, Chiva R (2013). Linking entrepreneurial orientation and firm performance: The role of organizational learning capability and innovation performance. J. Small Bus. Manag..

[40] Sakshi, Sharma C, Sharma S, Singh P, Khan IA (2021). Advanced attendance management systems: Technologies and applications. Edelweiss Appl. Sci. Technol..

[41] Arabeche Z, Soudani A, Brahmi M, Aldieri L, Vinci CP, Abdelli MEA (2022). Entrepreneurial orientation, organizational culture and business performance in SMEs: Evidence from emerging economy. Sustainability (Switzerland).

[42] Hidayat D, Abdurachman E (2022). The roles of gamification, knowledge creation, and entrepreneurial orientation towards firm performance. Int. J. Innov. Stud..

[43] Mozumdar L, Islam MA (2022). Business and family livelihood performance of Bangladeshi pond aquaculture entrepreneurs: Do business networks and entrepreneurial orientation matter?. Aquaculture.

[44] Forcadell FJ, Úbeda F (2022). Individual entrepreneurial orientation and performance: The mediating role of international entrepreneurship. Int. Entrep. Manag. J..

[45] Alam SS, Md Salleh MF, Masukujjaman M, Al-Shaikh ME, Makmor N, Makhbul ZKM (2022). Relationship between entrepreneurial orientation and business performance among Malay-owned SMEs in Malaysia: A PLS analysis. Sustainability (Switzerland).

[46] Manzano-García G, Ayala-Calvo JC (2020). Entrepreneurial orientation: Its relationship with the entrepreneur's subjective success in SMEs. Sustainability (Switzerland).

[47] Leonidou LC, Christodoulides P, Thwaites D (2016). External determinants and financial outcomes of an eco-friendly orientation in smaller manufacturing firms. J. Small Bus. Manag..

[48] Aftab J, Veneziani M, Sarwar H, Ishaq MI (2022). Organizational ambidexterity, firm performance, and sustainable development: Mediating role of entrepreneurial orientation in Pakistani SMEs. J. Clean. Prod..

[49] Frare AB, Beuren IM (2022). The role of green process innovation translating green entrepreneurial orientation and proactive sustainability strategy into environmental performance. J. Small Bus. Enterp. Dev..

[50] Dias C, Rodrigues RG, Ferreira JJ (2022). Linking natural resources and performance of small agricultural businesses: Do entrepreneurial orientation and environmental sustainability orientation matter?. Sustain. Dev..

[51] Bos-Brouwers HEJ (2010). Corporate sustainability and innovation in SMEs: Evidence of themes and activities in practice. Bus. Strategy Environ..

[52] Tiba S, van Rijnsoever FJ, Hekkert MP (2020). The lighthouse effect: How successful entrepreneurs influence the sustainability-orientation of entrepreneurial ecosystems. J. Clean. Prod..

[53] Hart SL, Dowell G (2011). Invited editorial: A natural-resource-based view of the firm: Fifteen years after. J. Manag..

[54] Kraus S, Burtscher J, Vallaster C, Angerer M (2018). Sustainable entrepreneurship orientation: A reflection on status-quo research on factors facilitating responsible managerial practices. Sustainability.

[55] Akula SC, Singh P (2021). Role of microfinance, women decision making and previous work experience in women entrepreneurship during Covid-19. Int. J. Econ. Finance Stud..

[56] Aragón-Correa JA, Hurtado-Torres N, Sharma S, García-Morales VJ (2008). Environmental strategy and performance in small firms: A resource-based perspective. J. Environ. Manag..

[57] Ab Wahab M (2021). Is an unsustainability environmentally unethical? Ethics orientation, environmental sustainability engagement and performance. J. Clean. Prod..

[58] Abdullah AAAH (2022). Sustainability orientation and environment sustainable performance: What is the role of corporate environmental responsibility?. J. Sustain. Sci. Manag..

[59] Danso A, Adomako S, Amankwah-Amoah J, Owusu-Agyei S, Konadu R (2019). Environmental sustainability orientation, competitive strategy and financial performance. Bus. Strategy Environ..

[60] Danso A, Adomako S, Lartey T, Amankwah-Amoah J, Owusu-Yirenkyi D (2020). Stakeholder integration, environmental sustainability orientation and financial performance. J. Bus. Res..

[61] Dias C, Gouveia Rodrigues R, Ferreira JJ (2021). Small agricultural businesses' performance—What is the role of dynamic capabilities, entrepreneurial orientation, and environmental sustainability commitment?. Bus. Strategy Environ..

[62] Eijdenberg EL, Sabokwigina D, Masurel E (2019). Performance and environmental sustainability orientations in the informal economy of a least developed country. Int. J. Entrep. Behav. Res..

[63] Rehman SU, Bresciani S, Yahiaoui D, Giacosa E (2022). Environmental sustainability orientation and corporate social responsibility influence on environmental performance of small and medium enterprises: The mediating effect of green capability. Corp. Soc. Responsib. Environ. Manag..

[64] Amankwah-Amoah J, Danso A, Adomako S (2019). Entrepreneurial orientation, environmental sustainability and new venture performance: Does stakeholder integration matter?. Bus. Strategy Environ..

[65] Roxas B (2021). Environmental sustainability engagement of firms: The roles of social capital, resources, and managerial entrepreneurial orientation of small and medium enterprises in Vietnam. Bus. Strategy Environ..

[66] Krejcie RV, Morgan DW (1970). Determining sample size for research activities. Edu. Psychol. Meas..

[67] Covin JG, Slevin DP (1989). Strategic management of small firms in hostile and benign environments. Strateg. Manag. J..

[68] Henseler J, Ringle CM, Sarstedt M (2015). A new criterion for assessing discriminant validity in variance-based structural equation modeling. J. Acad. Mark. Sci..

[69] Nitzl C, Roldan JL, Cepeda G (2016). Mediation analysis in partial least squares path modeling: Helping researchers discuss more sophisticated models. Ind. Manag. Data Syst..

[70] Temsas Z, Zemedu L, Kuma B, Mehari A (2021). Nexus between bank agriculture credit and economic development in Ethiopia: Ardl model approach. Int. J. Econ. Finance Stud..

[71] Martin-Tapia I, Aragon-Correa J, Rueda-Manzanares A (2010). Environmental strategy and exports in medium, small and micro-enterprises. J. World Bus..

[72] Vazquez-Carrasco R, Lopez-Perez M (2013). Small and medium-sized enterprises and corporate social responsibility: A systematic review of the literature. Qual. Quant. Int. J. Methodol..

[73] Relja R, Popović T, Tomić V (2016). The sustainability of tradition in the Dalmatian hinterland through green entrepreneurship. Int. J. Interdiscip. Environ. Stud..

[74] Weber H, Wiek A, Lang DJ (2020). Sustainability entrepreneurship to address large distances in international food supply. Bus. Strategy Dev..

[75] Satria H, Amar S, Wardi Y (2022). Impact of Nagari financial management on the performance of sustainable development in West Sumatra Province. Croat. Int. Relat. Rev..

