# Supplementary material for: The effect of environmental sustainability orientations and entrepreneurial orientations on the performance of greenhouses
Source: Sci Rep. 2024 Jan 24;14:2095. doi: 10.1038/s41598-024-52062-y (PMC10808209; doi:10.1038/s41598-024-52062-y)
Supplement: Supplementary file 1 — Supplementary Information. [file 41598_2024_52062_MOESM1_ESM.docx]

**Appendix:** All the items used for measuring variables

Environmental sustainability orientations’ components and items for measuring

| Component | Items |  |  |  |  |
| --- | --- | --- | --- | --- | --- |
| Awareness of environmental issues | Awareness of climate change and its impacts |  |  |  |  |
|  | Information on resources supplying drinking water |  |  |  |  |
|  | Adequate knowledge and information about different types of energy sources |  |  |  |  |
|  | Knowledge about environmental conservation by businesses |  |  |  |  |
|  | Awareness of the dimensions of sustainable development |  |  |  |  |
|  | Information on municipal wastes and garbage |  |  |  |  |
|  | Awareness of environmental conservation programs |  |  |  |  |
|  |  |  |  |  |  |
| Environmental practices | Responsibility for the environment along with job responsibility |  |  |  |  |
|  | Saving water and power consumption in the production unit |  |  |  |  |
|  | Participation in environmental conservation programs |  |  |  |  |
|  | Using technologies that reduce harmful environmental effects |  |  |  |  |
|  | Environmental protection as a production unit program |  |  |  |  |
|  | Communication with activists and environmentalists |  |  |  |  |
|  | Measures for waste recycling in the production unit |  |  |  |  |
|  | Dialogue with customers about environmental conservation |  |  |  |  |
|  | Educational programs for increasing environmental awareness |  |  |  |  |
|  |  |  |  |  |  |
| Commitment to environmental issues | Proud of working at the level of local communities |  |  |  |  |
|  | Adherence to environmental conservation as a job commitment |  |  |  |  |
|  | Thinking about the implementation of environmental protection measures |  |  |  |  |
|  | Attracting more customers owing to commitment to the environment |  |  |  |  |
|  | Environmental conservation at any expense as a goal of the working unit |  |  |  |  |

Scale: 1 = very low, 2 = low, 3 = moderate, 4 = high, 5 = very high

Entrepreneurial orientations’ components and items for measuring

| Component | Items |  |  |  |  |
| --- | --- | --- | --- | --- | --- |
| Tendency toward innovation | Encouraging employees to think and behave innovatively |  |  |  |  |
|  | Willingness to solve problems in new ways |  |  |  |  |
|  | Interest in finding new production ways |  |  |  |  |
|  | Earlier use of new technologies than competitors |  |  |  |  |
|  |  |  |  |  |  |
| Proactiveness in entrepreneurial orientation | Advancement in response to customer needs |  |  |  |  |
|  | Desire to identify and use new technologies |  |  |  |  |
|  | Anticipating future problems, needs or changes |  |  |  |  |
|  | Introducing new products to customers earlier than competitors |  |  |  |  |
|  | Using innovative methods earlier than others |  |  |  |  |
|  |  |  |  |  |  |
| Risk-taking | Willingness to boldly do risky things |  |  |  |  |
|  | Belief in producing high-risk products to keep competitiveness |  |  |  |  |
|  | Being risk-taking and welcoming new methods |  |  |  |  |
|  | Willingness to test and apply risky ideas |  |  |  |  |

Scale: 1 = very low, 2 = low, 3 = moderate, 4 = high, 5 = very high

Components of the performance of greenhouse units

| Component | Items |  |  |  |  |
| --- | --- | --- | --- | --- | --- |
| Increasing customers | increasing customer satisfaction versus previous years |  |  |  |  |
|  | Increasing customer loyalty versus the competitors’ customers |  |  |  |  |
|  | Improving trade relationships with customers versus previous years |  |  |  |  |
|  | increasing the number of customers versus previous years |  |  |  |  |
|  | | | | | |
| Increasing sales | increasing sales quantity in the last five years versus the competitors |  |  |  |  |
|  | increasing sales quantity over the last years |  |  |  |  |
|  | increasing market share of the unit versus the competitors |  |  |  |  |
|  | | | | | |
| Increasing profitability | increasing the ratio of profit to total sales versus the competitors |  |  |  |  |
|  | increasing total profit versus the competitors |  |  |  |  |
|  | increasing the ratio of profit to investment versus the competitors |  |  |  |  |

Scale: 1 = very low, 2 = low, 3 = moderate, 4 = high, 5 = very high
